# Bilateral pulmonary parenchymal metastasis from a low-grade appendiceal mucinous neoplasm

**DOI:** 10.1093/jscr/rjaf367

**Published:** 2025-06-05

**Authors:** John D M Cavaye, Bree D Stephensen, Jeffrey B Macemon

**Affiliations:** Department of General Surgery, Sunshine Coast University Hospital, 6 Doherty Street, Birtinya, Queensland, 4575, Australia; Department of General Surgery, Sunshine Coast University Hospital, 6 Doherty Street, Birtinya, Queensland, 4575, Australia; School of Medicine & Dentistry, Griffith University, Sunshine Coast Health Institute, 6 Doherty Street Birtinya, Queensland, 4575, Australia; Department of Thoracic Surgery, Sunshine Coast University Hospital, 6 Doherty Street, Birtinya, Queensland, 4575, Australia

**Keywords:** low-grade appendiceal mucinous neoplasm, LAMN, pulmonary metastasis

## Abstract

Low-grade appendiceal mucinous neoplasms, are the most common precursor lesions to pseudomyxomatous peritonei. They are relatively indolent in nature, with a “pushing” style of invasion. We present a case of a 50-year-old gentlemen who underwent staged video assisted thoracoscopic surgery for bilateral pulmonary metastasis secondary to a low-grade appendiceal mucinous neoplasm that had been resected 12 years prior. We highlight the under recognized rare etiology of distant metastasis without evidence of local spread, and therefore whether imaging of the chest should be considered as part of the surveillance protocol.

## Introduction

Appendiceal mucinous neoplasm (AMN) is a rare tumor arising from the appendix, found in <1% of appendicectomy specimens [1]. Originally described in 2003, they are characterized by a dilated and mucin filled appendix [2]. These tumors can further be classified as low-grade appendiceal mucinous neoplasm (LAMN) or high-grade appendiceal mucinous neoplasm (HAMN) based on cytological atypia and presence of infiltrative invasion [3]. LAMNs are the most common precursor lesions to pseudomyxoma peritonei (PMP), which occurs secondary to local infiltration, primarily after appendiceal perforation.

PMP is defined by its presence of mucinous ascites, peritoneal implants and omental caking [3]. Despite its slow progression, it causes debilitating symptoms and is ultimately fatal if left untreated, therefore, it is commonly treated with cytoreductive surgery (CRS) and hyperthermic intraperitoneal chemotherapy (HIPEC) [4].

Appendicectomy has commonly been the mainstay of treatment for LAMNs, with complete excision deemed curative. Higher staged lesions are then often surveilled to detect early progression to PMP [5, 6]. Classically, LAMN has been defined to have an inability to metastasise to distant areas of the body, secondary to its ‘pushing’ style of invasion [2]. Despite this, scarce literature reports have identified distant pulmonary metastasis, secondary to a LAMN [7–11].

We present a case of metastatic pleuropulmonary LAMN, 12 years after initial appendicectomy.

## Case report

A 50-year-old Caucasian male presented to the emergency department with chest wall pain after an accidental fall off his roof. Examination elicited no obvious injuries at time of his presentation, with normal vital signs. He had a background of laparoscopic appendicectomy followed by right hemicolectomy for synchronously identified appendiceal carcinoid tumor and mucocoele 12 years prior. His follow up ceased after he moved interstate to his current location.

**Figure 1:**
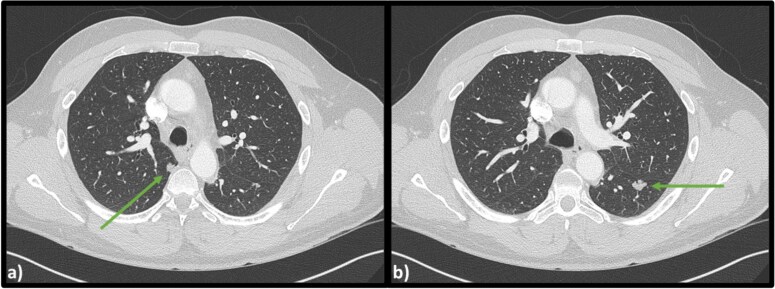
Axial CT scan of the chest indicating (a) lobulated lesion in apical segment of right lower lobe (9 × 10 × 10 mm) and (b) secondary lobulated lesion present in left lower lobe (15 × 11 × 14 mm).

Computed tomography (CT) scans revealed no injuries, however bilateral lobulated lower lobe pulmonary masses were identified measuring 15 × 11 × 14 mm on the left and 9 × 10 × 10 mm on the right (Fig. 1).

Respiratory function tests were normal, with a negative cryptococcus antigen. CT guided biopsy of his left lower lobe lesion was consistent with a low-grade mucin-producing tumor. It was likely this lesion was a metastasis from his previous appendiceal mucocele, and staged operative intervention to remove both pulmonary lesions was undertaken. Video assisted thoracoscopic surgery (VATS) resection of the left lower lobe superior segment revealed a well-circumscribed deposit of low-grade mucinous neoplasm. Histology comparing the pulmonary lesion with the original appendicectomy confirmed matching pathological appearance. Based on current nomenclature, his initial appendicectomy lesion would be defined as a LAMN with perforation, pT4b. Further right sided lower lobe superior segment pulmonary resection confirmed identical LAMN histology. The patient has since been discharged from hospital and has made a full recovery.

## Discussion

Cases of parenchymal pulmonary metastasis from appendiceal mucinous lesions are rare, first reported in 1962 where pulmonary metastasis from an appendiceal mucocele was identified post-mortem [7]. Mortman *et al.* later published three patients with pulmonary disease secondary to a mucinous lesion [8]. Two of these patients had appendiceal lesions that would be today classified as LAMN, and all three had evidence of disseminated abdominal PMP [8]. Further cases have been reported of parenchymal pulmonary lesions identified in patients that had previously undergone cytoreductive surgery for PMP [9, 10].

Appropriately, since 1962, the nomenclature to define such mucinous appendiceal lesions has evolved to the current tiered structure. The current system classifies mucinous lesions on a spectrum from LAMN through to invasive adenocarcinoma, based on features of invasion, cytological atypia and presence of signet ring cells [3]. However, in cases where low-grade lesions can metastasise distantly, authors have queried whether these cases are in fact adenocarcinomas due to the added threat they pose [8–10].

Most recently, Zhao *et al.* reported a case of LAMN with pulmonary metastasis at time of diagnosis [11]. The pulmonary lesion was initially diagnosed as an adenocarcinoma, however, next generation sequencing confirmed it had identical tumor mutations to the LAMN identified post hemicolectomy [11]. Notably, like our patient, their case had no evidence of disseminated PMP alongside the pulmonary disease. Our case supports this literature in identifying distant metastasis without evidence of localized “pushing” invasion. This may indicate that LAMNs have a potential for hematogenous spread, especially given our case demonstrates bilateral spread of disease. However, more investigation is required to identify the true propensity and mechanism in which these lesions manage to metastasize distantly.

Our case highlights the potential for disease metastasis long after initial diagnosis, suggesting more rigorous surveillance strategies for patients diagnosed with a LAMN need to be considered. There is limited consensus on appropriate guidelines to establish surveillance post LAMN diagnosis [5, 6, 12]. Two separate institutions have published their guidelines for postoperative surveillance using biochemical tumor markers alongside repeat abdominopelvic CT scans to assess for extension to PMP. Due to the risk of pulmonary metastasis, we recommend chest CT be added to surveillance protocols in patients with high-risk initial disease. To our knowledge, this is the first case of bilateral pulmonary metastasis from a LAMN.
